# Convalescent Plasma for COVID-19: A Single Center Prospective Experience with Serial Antibody Measurements and Review of the Literature

**DOI:** 10.3390/pathogens11090958

**Published:** 2022-08-23

**Authors:** Sonia N. Whang, Vivek D. Shah, Lu Pu, Parthiv Sheth, Gina N. Lucas, Joanne Lee, Michael Lee, Curtis Lacy, Penelope J. Duerksen-Hughes, Valeri Filippov, David J. H. Lee, Jennifer Veltman, Kanwaljeet Maken, Mark E. Reeves, Wesley Tait Stevens, Paul Herrmann, Huynh Cao, Eric Lau

**Affiliations:** 1 Department of Basic Science, School of Medicine, Loma Linda University, Loma Linda, CA 92354, USA; 2 Department of Medicine, Loma Linda University Health, Loma Linda, CA 92354, USA; 3 School of Medicine, Loma Linda University, Loma Linda, CA 92350, USA; 4 Division of Hematology and Oncology, Loma Linda University Health, Loma Linda, CA 92354, USA; 5 Department of Pulmonary and Critical Care, Loma Linda University Health, Loma Linda, CA 92354, USA; 6 Department of Pathology and Human Anatomy, School of Medicine, Loma Linda University, Loma Linda, CA 92354, USA

**Keywords:** convalescent plasma, COVID-19, antibody, biomarkers, kinetics, passive immunity, blood bank

## Abstract

Background: High-titer convalescent plasma given early for COVID-19 may decrease progression into a severe infection. Here, we reported a study of serial antibody measurements in patients who received CP at our center and performed a systematic review of randomized trials on CP. Methods: Our center participated in the Mayo Clinic Expanded Access Program for COVID-19 Convalescent Plasma. Patients diagnosed with COVID-19 by nasopharyngeal polymerase chain reaction at our center between April and August 2020 were included in the study if staffing was available for specimen collection. Through a colloidal gold immunochromatography assay, these patients’ IgM and IgG antibody responses were measured at baseline (Day 0) and after transfusion (Day 1, 2, etc.). Donor CP antibody levels were measured as well. Results: 110 serum specimens were obtained from 21 COVID-19 patients, 16 of whom received CP. The median time from developing symptoms to receiving CP was 11 days (range 4–21). In 9 of 14 (64%) cases where both recipient and donor CP antibody levels were tested, donor COVID-19 IgG was lower than that of the recipient. Higher donor antibody levels compared with the recipient (R = 0.71, *p* < 0.01) and low patient IgG before CP transfusion (*p* = 0.0108) correlated with increasing patient IgG levels from baseline to Day 1. Among all patients, an increased COVID-19 IgG in the short-term and longitudinally was positively correlated with improved clinical outcomes (ρ = 0.69, *p* = 0.003 and ρ = 0.58, *p* < 0.006, respectively). Conclusions: In a real-world setting where donor CP was not screened for the presence of antibodies, CP in donors might have less COVID-19 IgG than in recipients. An increase in patient antibody levels in the short term and longitudinally was associated with improved clinical outcomes.

## 1. Background

The use of passive immunotherapy with convalescent plasma (CP) to combat viruses spans over a hundred years. Until recently, the benefit of CP treatment has been largely based on observational studies [1,2]. Due to a lack of known efficacious agents early in the 2019 coronavirus disease (COVID-19) pandemic and retrospective data suggesting the benefit of CP in previous viral outbreaks, the Food and Drug Administration (FDA) approved an expanded access program (EAP) for COVID-19 CP therapy for critically ill COVID patients in March 2020 [3]. Since then, many studies have evaluated COVID-19 CP’s efficacy in improving clinical outcomes of patients with COVID, and the FDA has authorized an emergency use authorization (EUA) for CP.

Here we reported our single-center experience utilizing serial antibody measurements in patients who received COVID-19 convalescent plasma at Loma Linda University Medical Center (LLUMC) from April to August 2020. Specifically, we assessed the correlation between increased antibody levels and clinical outcomes. We reflected on our single-center experience considering published randomized-controlled efficacy data on COVID-19 CP and commented on how an effective CP program might be achieved in a future viral outbreak.

## 2. Materials and Methods

### 2.1. Design and Setting

Patients with a documented diagnosis of COVID-19 by nasopharyngeal polymerase chain reaction (PCR) at LLUMC from April to August 2020 were included in the study. LLUMC participated in the Mayo Clinic EAP for COVID-19 CP from 1 March to 30 September 2020, and obtained CP from our local blood bank, Lifestream. This prospective study was approved by the LLUMC Institutional Review Board (IRB approval #5200174) in June 2020.

All patients in the study were prospectively identified at the time of request to obtain CP and were intended to be recipients of CP. No more than one unit of CP (approximately 250 mL) was issued per requested patient, and CP was transfused within 24 h after thawing. All patients receiving CP at our institution were considered candidates for the study; however, patients were included in the study only if research staff were available at the time of CP infusion to collect blood and process the specimens. Some patients who were included in the study did not receive CP due to clinical improvement, medical futility as deemed by the treating team, or death. Clinical characteristics were identified in the patients, including age, time from symptoms to transfusion (T2CP), other treatments received, sequential organ failure assessment (SOFA) score at diagnosis [score range 1–5], Charlson Comorbidity Index (CCI) [index range 0–7], where higher SOFA and CCI numbers indicate higher morbidity. We also analyzed patient outcomes 30 days after CP intervention utilizing the World Health Organization (WHO) recovery clinical scale, which was defined as follows: 1–death, 2–invasive ventilatory support, 3–hospitalized with supplemental oxygen requirement, 4–hospitalized without supplemental oxygen requirement, 5–discharged without a full return to baseline physical function, 6–discharged with full return to baseline physical function [4].

### 2.2. Antibody Testing

**Figure 1:**
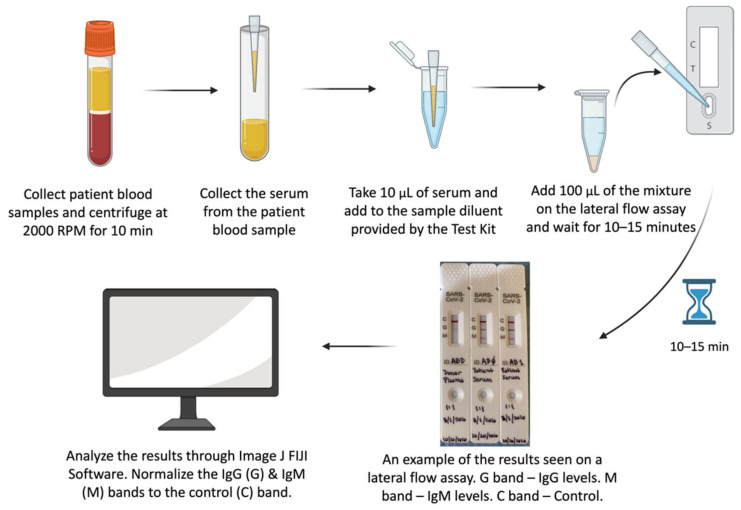
The steps outlined in the manufacturer’s protocol for “COVID-19 IgM/IgG Antibody Detection Kit; NBCP-0001”. IgG and IgM antibody responses were tested with lateral flow assays supplied by Nirmidas Biotech (Palo Alto, CA, USA). This lateral flow antibody assay uses the principle of colloidal gold immunochromatography and capture method to detect COVID-19 IgM and IgG antibodies from each sample. The flow assay detects the presence of antibodies to the S1 and receptor-binding domain (RBD) subunits of the spike protein. Patient blood specimens (3–5 cc) were collected and centrifuged at 2000 RPM for 10 min and stored at −20 °C until further use. The serum and plasma were separated and aliquoted in tubes before testing and were later stored at −80 °C. During testing, 10 µL of the serum or plasma was added to the sample diluent provided by the kit. 100 µL of this mixture was added to the sample well of the test card. Test results were observed after 10 min and recorded photographically. The photographed bands were quantified using Fiji ImageJ software. The IgG (G) and IgM (M) bands measured were normalized against the control band (C). The normalized densities of G and M were used as surrogates for antibody titers.

Antibody levels in both donor CP units and the recipients of CP were measured (Figure 1). Patients who received CP (CP^+^) had their blood samples drawn at baseline (Day 0) and after (Day 1, 2, etc.) CP transfusion. Antibody levels (Collection 1, 2, etc.) of patients who did not receive CP were similarly measured. For patients who did not receive CP (CP^−^), two or more blood samples were collected one to three days apart, and no collections beyond six days from the first blood collection were made. For most of the CP^+^ patients, daily blood samples were collected for the first week post-transfusion and then once a week thereafter until discharge or death. We performed correlation analyses on their antibody levels and calculated the correlation coefficient (R) and *p*-value using the Excel Data Analysis tool. Any associations between patients’ WHO clinical outcomes and COVID-19 antibody levels were analyzed using Spearman’s rank test. Statistical significance is indicated as follows: * *p* < 0.05, ** *p* < 0.01.

### 2.3. Logistic Regression Analysis

Among all patients, an increasing IgG level was positively correlated with more favorable clinical outcomes, as described in Section 3.2.3. Therefore, IgG level short-term changes (ratio of Day 1/Day 0) might be a useful predictor for clinical outcomes. As discussed in Section 4.2.3, CP treatment early in a disease’s progression may maximize its clinical efficacy. In addition, a high SOFA score is known to indicate higher morbidity.

Here we defined the WHO categories 1–3 as “Unfavorable Outcomes” and WHO categories 4–6 as “Favorable Outcomes” and proposed a prognostic score for CP transfusion using a simple formula: S = IgG score − SOFA score − T2CP score. The overall score (S) was a linear combination of three integer factors: (1) IgG score, that is, the IgG level short-term change (from Day 0 to 1) which was rounded up to the nearest integer; (2) SOFA score, which represented the baseline patient condition; and (3) the T2CP score, which was the duration (rounded to weeks) between symptom onset and the time of CP transfusion.

Using SPSS 25.0, a binary logistic regression was performed with the S-score as the independent variable and the binary clinical outcomes as dependent variables. The resulting log-odds (Z) of achieving a favorable outcome based on the S score are shown in the following equation:

Z = 2.27 × S + 7.70(1)

where log-odds Z = log(prob/(1−prob)), in which prob is the probability of having a “Favorable Outcome” and 1-prob is the probability of having an “Unfavorable Outcome”. When prob > 0.5 (Z > 0), the calculated outcome is assigned as “Favorable”, and when prob < 0.5 (Z < 0), the calculated outcome is assigned as “Unfavorable”.

### 2.4. Systematic Review

We conducted an extensive literature search of randomized controlled clinical trials (RCTs) from 1 January 2020 until the cutoff time of 5 May 2022 of the PubMed database. The following keywords were used: “convalescent plasma” AND (“randomized” OR “randomised”) AND “COVID-19”. The database search was conducted by three authors (E.L., P.S. and J.L.), who then extracted the data from the studies into a summary table. Only English-language papers that were randomized controlled trials involving COVID-19 were included. Preprints or trials without a clear power calculation for a primary endpoint were excluded.

## 3. Results

### 3.1. Cohort Characteristics

From March to September 2020, 129 patients hospitalized for COVID-19 at LLUMC received one unit of CP. 21 patients were included in the study and had COVID-19 antibody density levels serially measured. All patients received corticosteroids as part of their care. No patients had a history of hematologic malignancy, and no patients were actively in treatment for cancer. Moreover, no patients were COVID-19 vaccinated as vaccines were not yet commercially available during this period.

A total of 110 serum specimens were obtained from the 21 COVID-19 patients. 16 of 21 patients received CP, 2 of whom did not obtain baseline blood work prior to receipt of convalescent plasma. The median age was 59 (range 20–87), median CCI was 2.5 (range 0–7), and the median SOFA score at diagnosis was 3 (range 1–5). The majority (56%, 9/16) received CP while in the ICU, four of whom were on mechanical ventilation at the time of receipt. The remaining patients (44%, 7/16) received CP in medical wards. The average time from developing symptoms to receiving CP was 11 days (range 4–21 days). Moreover, we noted a moderately positive correlation between the illness duration from symptom to discharge and the patient’s WHO clinical scale (Rho (ρ) = 0.545, *p* = 0.011 *). At 30 days from receipt of plasma, 12 (75%) survived (eight patients had WHO scores of 6, and four had WHO scores of 5); three (19%) patients died (WHO score of 1); and one (6%) patient had a WHO score of 3 but died after 30 days.

Five patients did not receive CP, but blood specimens were still collected. The median CCI for this cohort was 1 (range 0–4), and the median SOFA score at diagnosis was 4 (range 2–6). At 30 days, three (60%) patients passed away (WHO score 1); one (20%) patient remained on mechanical ventilation (WHO score 2) and ultimately died, and one (20%) patient survived (WHO scale 5) (Table 1).

### 3.2. Antibody Analysis

#### 3.2.1. Donor vs. Recipient Antibody Levels

**Figure 2:**
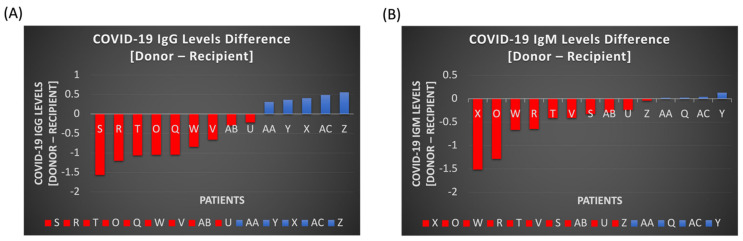
Differences in COVID-19 IgG (**A**) and IgM (**B**) antibody levels between donor and recipient (or patient). 9 (IgG) and 10 (IgM) donor plasmas’ titers displayed lower levels of COVID-19 antibodies compared to the recipients’, as depicted in the graphs in red. 5 (IgG) and 4 (IgM) patients’ convalescent plasma had higher COVID-19 antibody levels than the recipient’s endogenous levels, as shown in blue.

The antibody tests demonstrated that the donors’ COVID-19 antibody bands for IgG and IgM were often less dense than the recipients’ prior to CP treatment. The overall difference in donor and recipient antibodies of IgG and IgM band densities are visualized in Figure 2A,B, respectively. In regard to IgG alone, 9/14 (64%) patients had a lower IgG CP antibody density in the donor unit when compared to the recipient.

#### 3.2.2. Serial Patient COVID-19 IgG vs. Clinical Outcome

**Figure 3:**
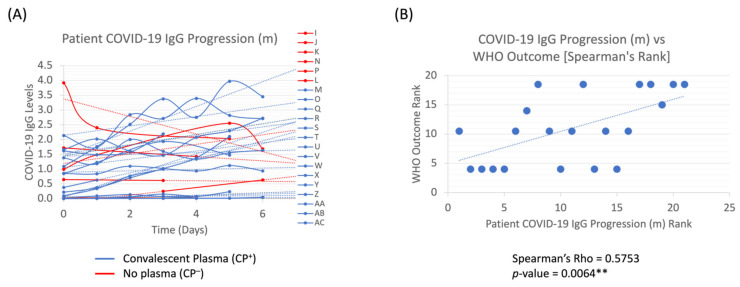
COVID-19 IgG Progression in Recipients. Longitudinal IgG progression (**A**) and Spearman’s correlation analyses between the trendline slopes (m) of the longitudinal progression and the WHO clinical scale (**B**) are depicted. *p*-value < 0.01 ** is statistically significant.

COVID-19 antibody levels were longitudinally measured throughout each patient’s hospitalization, and then the best fit trendline and its slopes (m) were calculated for the first 6 days to evaluate whether the IgG levels trended upwards (positive m-slope) or downwards (negative m-slope) throughout their stay (Figure 3A). Among all patients in the study, there was a positive correlation (Rho (ρ) = 0.5753, *p* = 0.0064 **) between increased COVID-19 IgG progression (m) and their clinical outcomes (Figure 3B).

#### 3.2.3. Patient COVID-19 IgG Ratio vs. Clinical Outcome

**Figure 4:**
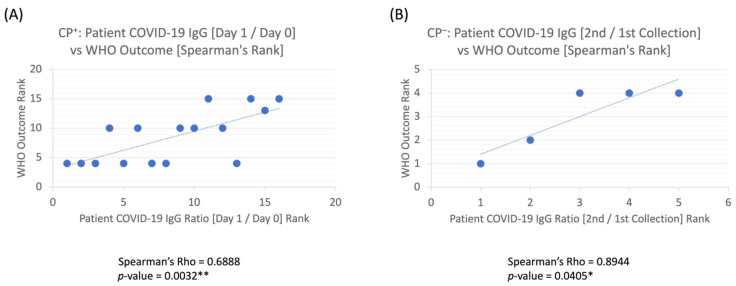
Spearman’s correlation coefficient analyses on (**A**) patient COVID-19 IgG [Day 1/Day 0] ratio vs. WHO clinical outcome: CP^+^ treated patients had a moderately positive correlation with improved outcomes. (**B**) Patient COVID-19 IgG [2nd/1st Collection] ratio vs. WHO clinical outcome: CP-untreated patients had a strong positive correlation to recovery outcome and showed statistical significance. *p*-values < 0.05 * & 0.01 ** are statistically significant.

COVID-19 antibody levels in the recipients were measured pre- and post-CP (Day 0 vs. Day 1), and a ratio of [Day 1/Day 0] patient IgG levels was calculated—whereby a proportion higher than one indicates an increase in COVID-19 IgG after plasma transfusion, and a ratio lower than one indicates a decrease in patient COVID-19 IgG levels after CP. Of the CP recipients with a baseline antibody density available, 10/14 (71%) had a WHO outcome between 4–6 at 30 days. Furthermore, higher antibody levels on the first day post-CP (Day 1) compared to baseline (Day 0), or [Day 1/Day 0] > 1, was positively correlated with improved clinical outcomes (Rho (ρ) = 0.6888, *p* = 0.0032 **) (Figure 4A). For the five patients who did not receive CP, one out of five (20%) had a WHO score between 4–6 at 30 days. Moreover, in CP^−^ patients, increasing antibody levels on the first two collections were highly positively correlated with better patient outcomes (Rho (ρ) = 0.8944, *p* = 0.0405 *) (Figure 4B).

#### 3.2.4. Patient COVID-19 IgG Ratio vs. Other Correlations

**Figure 5:**
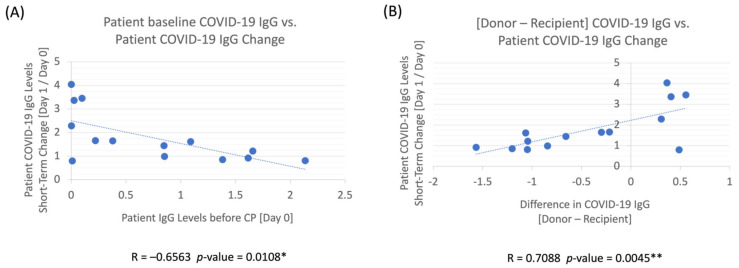
(**A**) Patient baseline COVID-19 IgG vs. patient COVID-19 IgG change (Day 1/Day 0): Patient’s COVID-19 IgG is less likely to increase after CP transfusion if the patient’s baseline IgG levels were already high before CP treatment. (**B**) Difference in COVID-19 IgG between donor and recipient vs. patient COVID-19 IgG change (Day 1/Day 0): Patient’s COVID-19 IgG levels post-transfusion compared to their baseline (Day 1/Day 0) increased as the difference between the donor and recipient’s baseline (Day 0) antibody levels was larger. *p*-values < 0.05 * & 0.01 ** are statistically significant.

Baseline COVID-19 IgG levels before CP treatment were shown to affect the IgG levels between Day 1 and Day 0. A high IgG baseline level was negatively correlated (R = −0.6563) with the changes in patient IgG levels between Day 1 and Day 0 (*p* = 0.0108 *) (Figure 5A). Additionally, to elucidate the absolute effect of donor antibody levels on the patient’s COVID-19 antibody levels, we examined the levels of donor’s IgG in relation to the patient’s IgG and its effect on the changes in the patient’s antibody levels between Day 1 and Day 0. The difference between donor and recipient IgG levels (Donor — Recipient) had a highly positive correlation (R = 0.7088) with the changes observed in the patient’s IgG ratios between Day 1 and Day 0, with *p* = 0.0045 ** (Figure 5B).

### 3.3. Logistic Regression Analysis

**Figure 6:**
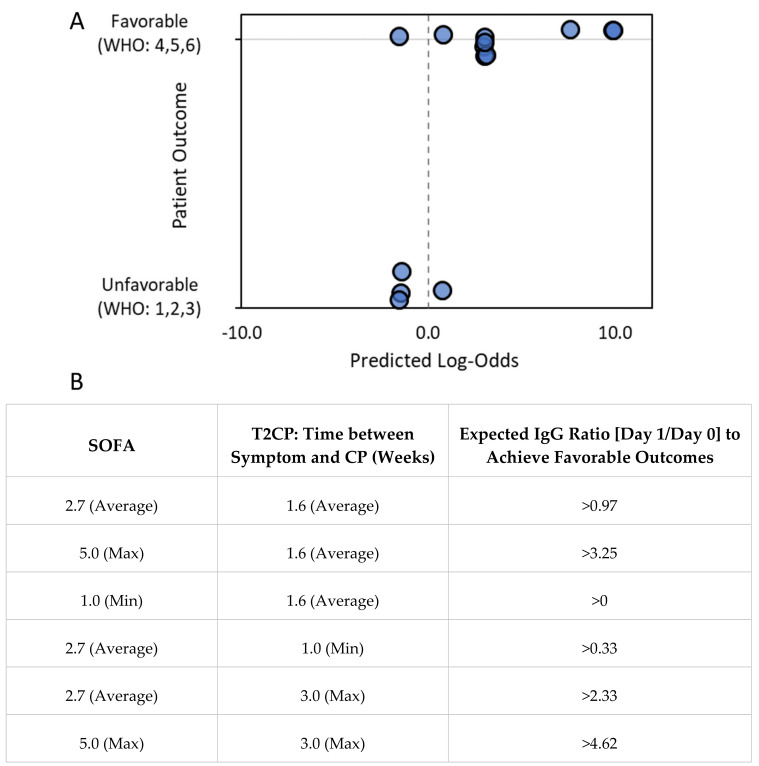
Higher IgG level increase from Day 0 to Day 1. Shorter time from symptom onset to CP transfusion and lower initial SOFA score were correlated with more favorable clinical outcomes. (**A**) Logistic regression analysis (Equation (1): Z = 2.27 × S + 7.70) showed that favorable clinical outcomes were positively correlated with the S-score, which was a linear combination of the IgG level increase from Day 0 to Day 1, time from symptom onset to CP transfusion, and the SOFA score. Regression coefficients are as follows: slope = 2.27, *p* = 0.03 (one-sided); constant, *p* = 0.032 (one-sided). The binary categorization accuracy based on the regression analysis was 85.7% (*n* = 14) when the binary categorization threshold was set at prob = 0.5 (log-odds = 0). Data markers were jittered for clarity. (**B**) Examples of calculations using Equation (1) for IgG level increase from Day 0 to Day 1 in situations with various time-to-treatment and SOFA score values to anticipate favorable outcomes (i.e., log-odds Z > 0).

Equation (1) was derived based on clinical analyses from 14 patients, where favorable clinical outcomes were positively correlated with the IgG level short-term increase (ratio of Day 1/Day 0) and negatively correlated with time from symptoms appearing to CP transfusion and SOFA scores. The average (±standard deviation, or std) IgG ratios, time from symptoms appearing to CP transfusion, and SOFA scores were: 1.79 ± 1.08, 11.36 ± 5.18 days, and 2.7 ± 1.4, respectively. The S-score has a range of −4 to 1 (with an average ± std of −2.1 ± 1.7). The calculated log-odds (Z) were from −1.4 to 10.0 (with an average ± std of 2.8 ± 4.0). The regression coefficients are as follows: slope = 2.27, *p* = 0.03 (one-sided); constant = 7.70, *p* = 0.032 (one-sided), and the regression analysis has an overall accuracy of 87.5% (Figure 6).

### 3.4. Literature Review

Our search yielded 236 reports, of which 24 peer-reviewed randomized controlled trials (RCT) utilizing CP in COVID-19 were screened for eligibility. One study was excluded due to a lack of power calculation for clinical outcomes [5]. Only 2 out of 23 published randomized clinical trials demonstrated an improved primary endpoint. The results are summarized in Table 2.

## 4. Discussion

### 4.1. Summary of Study Findings

Our study demonstrated that when donor CP units were not screened for presence of antibodies, a large proportion (64%) of donor units had lower antibody densities than recipients (Figure 2A,B). This may be explained as non-severe COVID-19 infected individuals, who are most likely to donate CP, have lower peaks of COVID-19 IgG antibodies than severely infected individuals [32]. Most patients have similar kinetics: rapid increase in antibody production with gradual drop-off followed by a plateau, and those with the more severe illness have a higher peak of total neutralizing antibody titers [33]. Hence, COVID-19 antibodies could rapidly degrade, which also suggested that optimal donation of CP is time-dependent [34].

We longitudinally tracked our patients’ antibody levels throughout each patients’ stay and noted in the full cohort (without regard to receipt of CP) that patients with a positive trendline (increasing IgG levels within the first 6 days) had more favorable outcomes than patients with a negative trendline (Figure 3B, *p* < 0.01**). Moreover, we analyzed the immediate effect of CP on the patient’s antibody levels by completion of the transfusion (i.e., by the next lab draw on Day 1). As CP infusion is expected to augment antibody to its highest level the day after transfusion but not long-term, we hypothesized that the ratio of antibody density between Day 1 and Day 0 would be a more direct measurement of the effect of CP on clinical outcomes. We found that patients who had an increased ratio of IgG antibody levels [Day 1/Day 0] trended towards better outcomes than those with lower ratios (Figure 4A, *p* = 0.0032 **). Our study suggests that an IgG increase both longitudinally and in the short-term between Day 1 and Day 0 can result in positive patient outcomes. Moreover, this kinetics conform with other studies in which SARS-CoV-2 Anti-RBD neutralizing IgG was associated with improved survival (hazard ratio of time to death 0.45), and anti-S1 + S2 IgA neutralizing antibodies were correlated with improved time to negative nasopharyngeal swab (hazard ratio 1.37) [35].

Additionally, a similar trend was seen with non-CP (CP^−^) recipients as those who received CP (CP^+^). For patients who had an increase in antibody density (ratio comparing 1st and 2nd blood sampling), WHO outcomes improved with increased ratio (Figure 4B; *p* < 0.05 *). However, the association based on coefficient was stronger with those who did not receive CP (Rho (ρ) = 0.8944) versus those who did (Rho (ρ) = 0.6888). This might suggest that patients who can boost their own response and have more rapid development of antibodies (based on higher ratios of second versus first blood collections) are more likely to have improved outcomes than patients who might not be able to boost their own immune response. Notwithstanding, the results from the CP^−^ group were derived from a low sample size and warrant a larger study to confirm the results.

Based on clinical findings, the logistic regression analysis result (Figure 6A,B) supported the conclusion that a higher IgG level increase (ratio of Day 1/Day 0), lower initial SOFA score, and earlier CP transfusion were overall helpful for favorable clinical outcomes. For a patient with an average to high SOFA score of 2.7–5 and symptoms of transfusion within 1–2 weeks, the initial IgG level increase [Day 1/Day 0] should be larger than 1–3 folds to expect a favorable clinical outcome.

A similar trend was additionally seen with non-CP (CP^−^) recipients as those who received CP (CP^+^). For patients who had an increase in antibody density (ratio comparing 1st and 2nd blood sampling), WHO outcomes improved with increased ratio (Figure 4B; *p* < 0.05 *). However, the association based on coefficient was stronger with those who did not receive CP (Rho (ρ) = 0.8944) versus those who did receive CP (Rho (ρ) = 0.6888). This suggested that patients who can boost their own response and have a more rapid development of antibodies (based on higher ratios of second versus first blood collections) are more likely to have improved outcomes than patients who may not be able to boost their own immune response.

These findings elucidated who benefits most from CP. The failure of CP in randomized trials to provide improved outcomes potentially reflects inappropriate patient selection. In our study, we noted a correlation with improved outcomes in patients with lower antibody levels (and thus higher [Day 1/Day 0] ratios) at the time of infusion (Figure 5A). CP treatment for patients without robust immunity to SARS-CoV-2 may lower mortality by providing passive immunity [1]. This premise was corroborated by an observational study in immunocompromised patients who had a solid organ or hematologic transplant, or active hematologic malignancy, presenting no detectable IgG against SARS-CoV-2 pre-CP; however, CP increased their antibody levels and offered positive outcomes based on the WHO recovery scale [36]. Elderly patients might also produce a less robust antibody response, as was seen in the negative correlation of age in a study in response to COVID-19 vaccines [37]. The positive results in the randomized INFANT-COVID-19 study, which only included elderly patients over the age of 75 or those who were over 65 with comorbidities, appeared to support that older patients are more likely to benefit from CP as well [11].

Our study had notable limitations. First, our study was small, limiting the statistical power and implications of our findings. During our study, many patients received CP overnight when staff could not process specimens rapidly, substantially limiting the number of subjects included in our study. The identity of the entire cohort of CP transfused patients outside of the study group was not available for comparison. Furthermore, although antibody measurements based on the quantification of antibody band density on lateral flow assay testing appeared to be a rational surrogate of antibody titers, we were unable to perform a study correlating these to neutralizing antibody titers. This led to uncertainty of the relationship of measured antibody densities with clinical trial definitions of high and low titer. In addition, the antibody quantification did not differentiate between S1 and RBD antibodies. Although we analyzed the outcomes of patients who did or did not receive CP, this assignment was not random and was influenced by selection bias. For example, the five patients included in this study who did not receive CP were initially intended to receive CP but did not after it was determined to be futile either due to rapid clinical decline or rapid clinical improvement.

### 4.2. Discussion of Optimizing Use of Convalescent Plasma in a Future Novel Viral Outbreak

Some studies have estimated approximately 1.67 million undescribed viruses to exist in mammals and birds, with up to half estimated to have the potential to cause a future pandemic [38]. The use of CP for COVID-19 is controversial. Multiple randomized controlled trials do not demonstrate the clinical benefit of CP as a treatment for COVID-19 (Table 2). The seventh update of the WHO guidelines on the treatment of COVID-19 strongly recommended against the use of CP outside of a clinical trial [39]. However, in the early periods of a future novel viral outbreak, CP may be one of the only treatment options available. CP appears to have modest efficacy and may need optimal conditions to demonstrate clinical benefits, such as high titer, early administration, and appropriately at-risk patients. Understanding how to optimally use our tools in fighting viral pandemics is crucial for future outbreaks.

#### 4.2.1. Optimizing Donations for Convalescent Plasma

To maximize the effectiveness of COVID-19 CP treatment, screening should be done on donor CP units for the presence of adequate titers. In the PLACID trial, which did not exclude any donor CP, nearly a third of units had undetectable antibody titers (1:20) with a median of 1:40. However, 83% of recipients had detectable antibodies (>1:90), indicating that the majority of patients received CP with lower titers than their endogenous production [8]. In the timeframe of our own study, our regional blood bank did not initially exclude units of CP based on the presence or absence of antibodies, and we observed similarly that 64% of the donor units appeared to have lower antibody density than recipients (Figure 2). Conversely, when the donor’s IgG concentration was higher than the recipient’s, the recipient’s IgG level also increased in the short term, as noted by a [Day 1/Day 0] > 1 (Figure 5B). To avoid futile treatment, a screening program in blood banks for measurably high antibody titers with neutralizing activity should be employed for donor CP units. Finally, securing donation in the early stage will be important because there is a time-dependent decrease in antibody titer [40]. It is generally accepted that the S-RBD IgG antibody level peaks approximately 28 days after the onset of symptoms and that there is a notable decline in donor antibody levels by 60 days post-symptoms onset [41].

#### 4.2.2. Optimal Candidates for Convalescent Plasma

Appropriate candidates for CP might include patients who are expected to produce poor antibody responses. Both the elderly and those with hematologic malignancies have been demonstrated to have suboptimal antibody responses to COVID-19 vaccination [37,42]. The INFANT-COVID19 trial demonstrated a reduced onset of respiratory failure in elderly patients given CP very early in their illness, and a large retrospective study suggested that patients with hematologic malignancies may derive survival benefits from CP treatment [11,43]. To date, such patient populations have not been the focus of most randomized clinical trials. Nonetheless, in concordance with the above, our study demonstrated that patients with lower titer pre-CP were more likely to increase their antibody titers after CP treatment [Day 1/Day 0] (Figure 5A) and more likely to have improved recovery (Figure 4A). Since donor units should be screened to ensure adequate antibody titers resulting in limited CP supply, optimal patient selection for CP would also become critical. To predict optimal patient selection, using data from the COMPILE meta-analysis, a tool called the Treatment Benefit Index was formulated. This tool offers a means of identifying patients who would benefit from CP, namely those with pre-existing conditions (diabetes, cardiovascular, and pulmonary diseases), blood type A or AB, and, as discussed above, those early in their illness [44].

#### 4.2.3. Optimal Timing of Treatment and Delivery of Convalescent Plasma

Utilizing CP as treatment early in the disease may maximize its clinical efficacy. In a national U.S. retrospective COVID-19 study, early CP treatment (within 3 days of diagnosis) was associated with improved clinical outcomes (22.2% vs. 29.5% 30-day mortality). Furthermore, those who received high-titer CP and were not intubated had a 14.2% 30-day mortality compared with 40.5% in those who were mechanically ventilated, indicating better outcomes if CP is administered before patients’ symptoms become severe and mechanical ventilation is necessary [45]. Based on this study, the Infectious Disease Society of America has suggested CP be administered within 3 days from diagnosis to be considered early administration [46]. In the INFANT-COVID-19 trial, patients symptomatic for less than 72 h received CP or placebo, and a reduction in severe illness was noted with CP (16% vs. 31% mortality, respectively *p* = 0.03) [11]. Although limited by selection bias, this potentially suggests that those who are early in their diagnosis and not critically ill may benefit most from CP. Moreover, it was possible that the negative results of multiple RCTs of COVID-19 CP were in part related to their late administration of CP (Table 2). In concordance with these studies, the FDA’s EUA for CP was amended in March 2021 to authorize high-titer CP to only be utilized early in the disease course (prior to the onset of respiratory failure requiring mechanical ventilation). The FDA also recognized that the therapeutic window might be longer in patients with impaired humoral immunity [47].

Another study has additionally shown that monoclonal antibodies (mAbs) against COVID-19 may have efficacy as post-exposure prophylaxis in a high-risk nursing home setting, suggesting a possible role for CP in the same clinical scenario prior to any symptom onsets. The COVID-19 neutralizing IgG1 mAb, Bamlanivimab, was shown to reduce viral replication and entry into airways in preclinical trials. Moreover, the BLAZE-2 trial evaluated the use of post-exposure prophylaxis with Bamlanivimab at nursing facilities, where each had at least one confirmed case of COVID-19. The treatment versus placebo group had a reduction in viral detection (15.2% vs. 19.9%) [48]. These studies highlight the use of antibody treatment as prophylaxis to reduce local outbreaks in congregate living arrangements or for immunocompromised individuals. Following the model of mAbs, clinical trials could be designed to administer high-titer CP as post-exposure prophylaxis, especially in vulnerable individuals in a viral outbreak, before monoclonal antibody treatments are developed.

Another potential strategy for early administration of CP might be identifying vulnerable patients with humoral dysfunction in the emergency department. The SIREN-C3PO study suggests the feasibility of providing high-titer CP to patients in the emergency department with an onset of symptoms of less than 7 days [20]. This study did not demonstrate the clinical benefit of CP, likely due to its younger, healthier population with a more robust immune response, and hence was terminated early. A future clinical trial or practice in a future novel viral outbreak could enroll patients expected to have poor humoral responses in the emergency department of an academic center, where eligibility criteria would be reviewed and CP offered. However, this would exclude a large population of individuals without nearby access to an academic center.

## 5. Conclusions

Our single-center experience highlights the importance of appropriate screening of CP for antibody titers as well as the population who may most benefit from this treatment. We noted that when COVID-19 IgG levels increased throughout the hospitalization, outcomes were more promising than when antibody levels had a general decline (Figure 3B). Similarly, those who had higher post-CP transfusion IgG [Day 1/Day 0] ratios had improved outcomes based on the WHO scale (Figure 4A,B). In exploring who might benefit from CP, we found that those who had low antibody levels at baseline had a higher increase in COVID-19 antibody levels post-treatment [Day 1/Day 0] (Figure 5A), in line with data demonstrating that those with poor humoral responses such as the elderly or with hematologic malignancies may benefit the most. Therefore, recipients should be screened to identify who is likely to benefit from an increase in antibody levels and determine whether there is a presence of immune response. If the patients already have an immune response, then receiving extra IgG through passive immunity might not provide much therapeutic benefit. If the patient does not have an immune response, then CP could render some benefit. More importantly, a screening program for CP titers would be necessary to ensure that the IgG amount is sufficient to have a therapeutic impact on the recipient. CP remains one of the earliest treatments available, and clinical trials should be designed to provide high titer CP early to patients, especially in those with poor humoral responses.

## Figures and Tables

**Table 1:** Description and Characteristics of the Patients in the Study.

Age/Sex	Charlson Comorbidity Index (CCI)	SOFA Score	Additional Treatments Received	Location of CP Receipt	Symptoms → CP Infusion (Time in Days)	Donor Ab Relative Densities (Donor IgG vs. Recipient IgG)	Recipient Ab Trend Day 0 → Day 1	WHO Outcome (30 Days after Intervention)
Recipients of Convalescent Plasma (CP+)								
74/F	3	4	Remdesivir, dexamethasone	ICU	9	IgG: D < R (1.08 vs. 1.71) IgM D < R (0.12 vs. 1.21)	Similar/Increase IgG (1.71 to 1.73) IgM (1.21 to 1.21)	3
57/M	2	1	Remdesivir, dexamethasone, tocilizumab	Ward	14	IgG: D < R (0.049 vs. 1.61) IgM: D < R (0.412 vs. 0.74)	Decrease IgG (1.61 to 1.48) IgM (0.74 to 0.65)	6
87/M	6	3	Remdesivir, dexamethasone, mechanical ventilation	ICU	17	IgG: D < R (0.18 vs. 1.38) IgM: D < R (0.23 vs. 0.67)	Decrease IgG (1.38 to 1.18) IgM (0.67 to 0.48)	1
62/F	0	1	Dexamethasone	Ward	11	IgG D < R (0.009 vs. 0.85) IgM D < R (0.22 vs. 0.88)	Similar/Decrease IgG (0.85 to 0.84) IgM (0.88 to 0.80)	5
63/M	3	2	Dexamethasone	Ward	14	IgG: D < R (0.026 vs. 1.1) IgM: D < R (0.06 vs. 0.47)	Increase IgG (1.1 to 1.8) IgM (0.47 to 0.72)	5
37/M	0	4	Dexamethasone	ICU	15	IgG D > R (0.43 vs. 0.027) IgM D < R (0.15 vs. 1.67)	Increase IgG (0.027 to 0.091) IgM (1.67 to 2.02)	5
25/M	0	4	Tocilizumab, Dexamethasone	Ward	12	IgG D < R (0.19 vs. 0.85) IgM D < R (0.02 vs. 0.44)	Increase IgG (0.85 to 1.23) IgM (0.44 to 0.58)	5
59/M	6	4	Tocilizumab, dexamethasone, mechanical ventilation	ICU	17	IgG: D < R (0.61 vs. 1.66) IgM D > R (0.35 vs. 0.33)	Increase IgG (1.66 to 2.02) IgM (0.33 to 0.49)	1
40/F	0	1	Dexamethasone, remdesivir	Ward	21	IgG D < R (0.089 vs. 0.38) IgM D < R (0.001 vs. 0.29)	Increase IgG (0.38 to 0.62) IgM (0.29 to 0.36)	6
42/M	0	1	Dexamethasone	ICU	6	IgG D < R (0.004 vs. 0.221) IgM D < R (0.012 vs. 0.25)	Increase IgG (0.22 to 0.37) IgM (0.25 to 0.32)	6
59/F	4	5	Dexamethasone, remdesivir	Ward	6	IgG D > R (0.31 vs. 0.0034) IgM D > R (0.047 vs. 0.029)	Increase IgG (0.0034 to 0.0077) IgM (0.026 to 0.030)	5
76/M	7	3	Remdesivir, tocilizumab, anakinra, dexamethasone	ICU	7	IgG D > R (0.36 vs. 0.001) IgM D > R (0.13 vs. 0.009)	Increase IgG (0.0019 to 0.0079) IgM (0.0096 to 0.0106)	6
55/F	1	2	Dexamethasone, tocilizumab	Ward	6	IgG D > R (0.65 vs. 0.09) IgM D < R (0.06 vs. 0.1)	Increase IgG (0.098 to 0.34) IgM (0.10 to 0.22)	6
79/F	5	3	Remdesivir, dexamethasone, mechanical ventilation	ICU	4	IgG D > R (0.49 vs. 0.009) IgM D > R (0.05 vs. 0.01)	Mixed IgG (0.009 to 0.007) IgM (0.011 to 0.023)	1
CP given (CP^+^); no baseline collection								
21/M	0	4	Hydroxychloroquine, methylprednisolone, tocilizumab, mechanical ventilation	ICU	11	N/A	Increase IgG (0.25 to 0.63) IgM (0.20 to 0.32)	6
77/M	5	4	Dexamethasone	ICU	6	N/A	Mixed IgG (0.016 to 0.059) IgM (0.071 to 0.058)	6
Patients who did not receive convalescent plasma (CP^−^)								
53/F	1	2	Hydroxychloroquine, methylprednisolone, hyperbaric oxygen therapy, mechanical ventilation	N/A	-	No CP received	Decrease IgG (1.71 to 1.42) IgM (0.65 to 0.50)	1
57/M	2	6	Hydroxychloroquine, methylprednisolone, tocilizumab, plasma exchange, mechanical ventilation	N/A	-	No CP received	Mixed IgG (0.036 to 0.025) IgM (0.47 to 0.57)	1
34/M	0	3	Hydroxychloroquine, methylprednisolone, anakinra, tocilizumab, plasma exchange, mechanical ventilation	N/A	-	No CP received	Decrease IgG (0.65 to 0.62) IgM (1.04 to 0.98)	2
40/M	0	4	Dexamethasone, tocilizumab,	N/A	-	No CP received	Decrease IgG (3.91 to 2.39) IgM (2.1 to 1.69)	1
74/F	4	4	Dexamethasone, tocilizumab	N/A	-	No CP received	Increase IgG (0.99 to 1.48) IgM (0.38 to 0.61)	5

Abbreviations: ICU—intensive care unit. N/A—not applicable.

**Table 2:** Twenty-three Randomized Controlled Trials on COVID-19 CP.

Author, Date of Publication, Name (Country of Study)	Study Design	Study Arms, Number of Participants	Median Age of Interventional Arm. % of Patients with Cancer, Immunocompromised, or on Immunosuppressants (%/% in Each Study Arm, If Specified)	Use of Glucocorticoid, Oxygen (%/% in Each Study Arm). Other Concomitant Therapies during Trial	Median Days from Symptom Onset to Randomization to CP	SARS-CoV-2 Antibody Titers and Presence of Antibody at Baseline	Median CP Volume. CP Antibody Titer	Primary Endpoint, Power Calculation, and Comments
Li et al. June 2020 [6] (China)	Open-label, single blind (investigator), multicenter RCT	N = 103 hospitalized patients; arm 1: CP + SOC (52); arm 2: SOC (51)	70 years (IQR 62, 80); cancer (5.8% vs. 0%); unvaccinated for SARS-CoV-2 (100%)	Glucocorticoid used (45.7% vs. 32.7%); O_2_ used (98% vs. 98%); other: antiviral medications, antibacterial medications, human immunoglobulin, Chinese herbal medicines	27 days (IQR 22, 39)	N/A	200 mL (IQR 200, 300); not clearly containing high-titer CP (At least 1:640 (S-RBD specific IgG antibody titer) [7]	No difference in time to clinical improvement (discharged alive or reduction of 2 points on a 6-point disease severity scale) within 28 days: 28 days vs. indeterminate (HR 1.4, 95% CI 0.79–2.49, *p* = 0.26); underpowered (103 recruited of 200 needed patients to achieve 80% power, 2-sided alpha 0.05, effect size 8 days).
Agarwal et al. October 2020 PLACID trial [8] (India)	Pragmatic open-label multicenter RCT	N = 464 hospitalized patients; arm 1: CP + SOC (235); arm 2: SOC (229)	52 years (IQR 42, 60); cancer history (0.4% vs. 0%); unvaccinated for SARS-CoV-2 (100%)	Glucocorticoid used (62% vs. 63%); mean FiO_2_ required to maintain SpO_2_ (39% vs. 37.4%); other: hydroxychloroquine, remdesivir, lopinavir/ritonavir, heparin products, azithromycin and “other antibiotics”, tocilizumab	8 days (IQR 6, 11)	Detectable neutralizing antibody titer of 418 tested patients: 185/215 (86%) (CP arm) 163/203 (80%) (SOC arm)	400 mL (approximate); no high-titer CP (median neutralizing antibody titer 1:40; IQR 1:30, 1:80)	No reduction in severe infection within 28 days or all-cause mortality at 28 days in intention-to-treat analysis: 19% vs. 18% (HR 1.07, 95% CI 0.73 to 1.58); powered (464/452 participants needed for 80% power, alpha 0.05, sidedness not specified, effect size 9%).
Simonovich et al. November 2020 PlasmAr trial [9] (Argentina)	Double-blinded, multicenter, placebo-controlled RCT	N = 333 hospitalized patients; arm 1: CP + SOC (228); arm 2: placebo (Saline) + SOC (105)	62.5 years (IQR 53, 72.5); hematological cancer (1.8% vs. 2.9%); solid cancer (10.1% vs. 10.5%); immunosuppressants (2.6 vs. 2.9%); unvaccinated for SARS-CoV-2 (100%)	Glucocorticoid used (91.7% vs. 96.2%); O_2_ used (90.4% vs. 88.6%); other: lopinavir-ritonavir, tocilizumab, ivermectin, hydroxychloroquine.	Not specified	Of 215 tested patients: median 1:50 (0–1:800) with detectable SARS-CoV-2 antibody titers in 80/145 (55%) (CP arm) vs. median 1:50 (0–1:1600) with detectable SARS-CoV-2 antibody titers in 36/70 (51%) (SOC arm)	500 mL (IQR 415, 600); high-titer CP [Median neutralizing antibody titer (COVIDAR IgG ELISA) 1:3200; IQR 1:800, 1:3200. Considered high titer by FDA [10]]	No difference in clinical status at 30 days (including death, invasive ventilatory support, hospitalized with or without oxygen requirement, discharged with or without return to baseline physical function): OR 0.81, 95% CI 0.5–1.31, *p* = 0.39; powered (333/333 enrolled needed for 80% power, 2-sided alpha 0.05, effect size of odds ratio of 1.8 for CP compared to placebo).
Libster et al. January 2021 INFANT-COVID trial [11] (Argentina)	Double-blinded, multicenter, placebo-controlled RCT	N = 160 mixture of hospitalized and geriatric institution patients Arm 1: CP (80) Arm 2: Placebo (Saline) + SOC (80)	76.4 years (SD ± 8.7); cancer in remission (5% vs. 2%); unvaccinated for SARS-CoV-2 (100%)	No glucocorticoid used. No baseline use of O_2_; other: none	1.65 days (SD ± 0.57 days; all patients were given CP within 72 h of symptoms)	N/A	250 mL (approximate); high-titer CP (IgG titer against SARS-CoV-2 spike protein (COVIDAR IgG ELISA) at least 1:1000; considered high titer by FDA [10])	Reduced progression of severe respiratory disease in intention-to-treat analysis: RR 0.52, 95% CI 0.29–0.94, *p* = 0.03; underpowered (160 enrolled of 210 needed to have 80% power, 2-sided alpha 0.05, effect size of 40% relative risk reduction). Study was stopped early due to finding clinical benefit. INFANT-COVID-19 notable limitations: (1) “Progression to severe respiratory disease” is used as a surrogate for mortality. (2) The study was stopped early due to positive findings. (3) Some authors have calculated a fragility index of 1 for the positive primary endpoint indicating poor robustness of this finding [12].
Balcells et al. March 2021 [13] (Chile)	Open-label, single-center RCT	N = 58 hospitalized patients; arm 1: early CP (28); arm 2: deferred CP to be given at time of clinical deterioration (PaO_2_/FiO_2_ < 200 or still requiring hospitalization for symptomatic COVID >7 days after enrollment) (30)	64.3 years (range 33–92); cancer (3.6% vs. 10%); immunosuppressants (14.3% vs. 10%); unvaccinated for SARS-CoV-2 (100%)	Glucocorticoid used (82.1%, 66.7%); O_2_ used (82.1%, 76.7%); other: tocilizumab, hydroxychloroquine, lopinavir/ritonavir, thromboprophylaxis, anticoagulation	Early group: 5 days from symptom onset at enrollment (IQR 4, 7) Deferred group: 6 days (IQR 4, 7)	Of 46 tested patients: median 1:400 (1:100–1:800) with positive SARS-CoV-2 IgG assay in 7/26 (27%) (CP arm) vs. median 1:400 (1:100–1:3200) with positive SARS-CoV-2 IgG assay in 5/20 (25%) (SOC arm)	400 mL (approximate) Titers not reported	No benefit in composite primary outcome of mortality, hospitalization over 14 days, or need for mechanical ventilation in intention-to-treat analysis: 32.1% vs. 33.3% (OR 0.95, 95% CI 0.32–2.84, *p* > 0.99); powered (58/58 enrolled for 80% power, 2-sided alpha 0.05, and effect size of absolute risk reduction of 35%).
RECOVERY Collaborative Group 2021 [14] (United Kingdom)	Open-label, multicenter RCT	N = 11,558 hospitalized patients; arm 1: CP + SOC (5795); arm 2: SOC (5763)	63.5 years (SD ± 14.7), no patients specified with cancer or immunosuppressant medication; unvaccinated for SARS-CoV-2 (100%)	Glucocorticoid used (93%, 92%); O_2_ used (87%, 87%); other: hydroxychloroquine, azithromycin, lopinavir/ritonavir, aspirin, colchicine	9 days (IQR 6, 12)	3779/5795 (65%) positive for SARS-CoV-2-IgG antibodies (CP arm) vs. 4103/5763 (71%) positive for SARS-CoV-2-IgG antibodies (SOC arm)	550 mL (range 400–700); high-titer CP (EUROIMMUN IgG ELISA targeting spike glycoprotein cutoff of 6; correlating to neutralizing titers of 1:100)	No difference in 28-day mortality in intention-to-treat analysis: 24% vs. 24% (RR 1.0, 95% CI 0.93–1.07, *p* = 0.95); powered (11,558 enrolled with 11,000 needed to achieve 90% power with 2-sided alpha 0.01 and effect size of absolute risk reduction of 20%)
Al Qahtani et al. May 2021 [15] (Bahrain)	Open-label, single-center RCT	N = 40 hospitalized patients; arm 1: CP + SOC (20); arm 2: SOC (20)	50.7 years (SD ± 12.5); no patients specified with cancer or immunosuppressant medication; unvaccinated for SARS-CoV-2 (100%)	Glucocorticoid used (5%, 20%); O_2_ used (100%, 100%); other: hydroxychloroquine, lopinavir/ritonavir, ribavirin, azithromycin, peginterferon, tocilizumab, methylprednisolone, antibiotics, anticoagulation, proton pump inhibitor, angiotensin-converting enzyme inhibitor/angiotensin receptor blocker	N/A	N/A	400 mL Titers not reported	No difference in primary outcome of requirement of ventilation (non-invasive or mechanical) in intention-to-treat analysis: RR 0.67, 95% CI 0.22–2.0, *p* = 0.72; underpowered (40 of 1644 enrolled to achieve 90% power, 2-sided alpha 0.05, effect size risk ratio of 0.7).
Gharbharan et al. May 2021 ConCOVID trial [16] (Netherlands)	Open-label, multicenter RCT	N = 86 hospitalized patients Arm 1: CP + SOC (43) Arm 2: SOC (43)	63 years (IQR 56, 74); cancer (12% vs. 7%); immunodeficiency (12% vs. 14%) Unvaccinated for SARS-CoV-2 (100%)	Use of Glucocorticoid not recorded; O_2_ used (16%; 2%); other: chloroquine, azithromycin, lopinavir/ritonavir, tocilizumab, anakinra	9 days (IQR 7, 13)	Of 66 tested patients: PRNT50 titer median 1:80 (IQR 1:20–1:640) with SARS-CoV-2 IgG positive in 26/32 (81%) (CP arm) vs. 1:320 (1:20–1:1280) with SARS-CoV-2 IgG positive in 27/34 (79%) (SOC arm)	300 mL high-titer CP (median PRNT50 titer 1:640; IQR 1:320, 1:1280)	No difference in 60-day mortality: 14% vs. 26% (OR 0.95, 95% CI 0.20–4.67, *p* = 0.95); underpowered (86/426 enrolled needed for 80% power, 2-sided alpha 0.05, effect size 30%). Halted early due to similar Ab titers in recipients and donor units.
O’Donnell et al. July 2021 [17] (New York and Rio de Janeiro)	Double-blinded, multicenter, placebo-controlled RCT	N = 223 hospitalized patients; arm 1: CP + SOC (150); arm 2: standard plasma + SOC (73)	60 years (IQR 48, 71); HIV (3% vs. 0%); no patients otherwise specified with cancer or immunosuppressants; unvaccinated for SARS-CoV-2 (100%)	Glucocorticoid used (81% vs. 82%); O_2_ used (94% vs. 93%); other: Remdesivir, hydroxychloroquine, antibacterial agent.	10 days (IQR 7, 13)	N/A	200–250 mL mixture of high and non-high titer CP (median neutralizing antibody titer 1:160; IQR 1:80–1:320)	No difference in improvement of clinical scale at 28 days in intention-to-treat analysis: OR 1.5, 95% CI 0.83–2.68, *p* = 0.18; powered (223 enrolled with 219 needed to achieve 82% power, 1-sided Mann-Whitney test at level of 15%, to detect odds ratio 1.7)
Bennet et al. July 2021 [18] (USA)	Double-blinded, single-center, placebo-controlled RCT	N = 74 hospitalized patients; arm 1: CP + SOC (59); arm 2: standard plasma + SOC (15)	67 years (SD ± 15.8); immunosuppressants (6.8% vs. 13.3%); unvaccinated for SARS-CoV-2 (100%)	Glucocorticoids used (62.7% vs. 53.3%); O_2_ used (74.5% vs. 60%); other: remdesivir, tocilizumab, hydroxychloroquine, sarilumab	9 days (IQR 6, 18)	Median titers: ~1:210 (IQR 1:90–1:350) (CP arm) vs. ~1:310 (IQR 1:140–1:360) (SOC arm)	480 mL (approximate) high-titer CP (median PRNT titer 1:526; IQR 1:359,1:786)	No significant difference between study groups was observed for ventilator-free days through 28 days in intention-to-treat analysis: median days 28 vs. 28 (*p* = 0.86); underpowered (74/500 enrolled to achieve 90% power, 2-sided alpha 0.05, effect size 2.5 days)
Sekine et al. July 2021 PLACOVID trial [19] (Brazil)	Open-label, single-center RCT	N = 160 outpatients; arm 1: CP + SOC (80); arm 2: SOC (80)	59 years (IQR 48, 68.5); no patients specified with cancer or immunosuppressant medication; unvaccinated for SARS-CoV-2 (100%)	Glucocorticoids used (98.8% vs. 98.8%); O_2_ used (100% vs. 99%); other: “antibacterials”	10 days (IQR 8, 12)	Neutralizing antibody titers >1:80 present in 133/160 (83.1%) at baseline, with median 1:1280 (IQR 1:320–1:2560)	600 mL high-titer CP (median neutralizing antibody titer 1:1280; IQR 1:320,1:2560)	No significant difference in clinical improvement at day 28 in intention-to-treat analysis: 61.3% vs. 65% (Risk difference −3.7%, 95% CI −18.8% to 11.3%); powered (160/160 enrolled to achieve 80% power, 2-sided alpha 0.05, effect size of 20% absolute difference).
Korley et al. August 2021 SIREN-C3PO trial [20] (USA)	Open-label, multicenter, single-blind (patients) randomized placebo-controlled trial	N = 511 hospitalized patients; arm 1: CP + SOC (257); arm 2: placebo (saline colored with parenteral multivitamin concentrate; 254)	54 years (IQR 47, 62); active Cancer (0.8 vs. 0.8%); immunosuppressants (12.8% vs. 6.7%); argan transplant recipients (1.9% vs. 0%); unvaccinated for SARS-CoV-2 (100%)	No glucocorticoids used. No baseline use of O_2_	4 days (IQR 2, 5)	N/A	250 mL (approximate) high-titer CP (median neutralizing antibody titer 1:641 (IQR not reported; study utilized PRNT50 of 1:250 or more)	No significant difference in disease progression at day 15 in intention-to-treat analysis: 30% vs. 31.9% (risk difference 1.9%; 95% CI −6.0 to 9.8; posterior probability of superiority 0.68); underpowered (511 of 900 patients to achieve 85% power, posterior probability of 0.975 selected to coincide with one-sided alpha 0.25; effect size 10%. Trial enrollment halted after second planned interim analysis showing that a priori stopping threshold for futility was reached (posterior predictive probability of success of 0.042).
Devos et al. August 2021 DAWn-plasma trial [21] (Belgian)	Open-label, multicenter RCT	N = 483; arm 1: CP + SOC (320); arm 2: SOC (163)	62 years (SD ± 14); active cancer (6.3% vs. 5.6%); chronic systemic corticosteroids (8.5% vs. 10.6%); other immunosuppressive therapy (6.9% vs. 10.6%); HIV/AIDS (1% vs. 0%); unvaccinated for SARS-CoV-2 (100%)	Glucocorticoids used (65% vs. 69.1%) O_2_ used (88.8% vs. 87.1%) (Excluded all patients on mechanical ventilation at baseline) Other: hydroxychloroquine, remdesivir, tocilizumab, lopinavir/ritonavir, other “antiviral drugs”, antibiotics, antifungal treatment, anticoagulation	7 days (IQR 4, 10)	Of 163 tested patients, 33/110 (30%) had neutralizing serum titers > 1:320 (CP arm) vs. 14/53 (26%) had neutralizing serum titers > 1:320 (SOC arm)	884 mL (IQR 806, 906) mixture of high and non-high titer CP (neutralizing antibody titer at least 1:320 received by 80% of patients)	No significant difference in number of patients alive without mechanical ventilation at day 15: 83.7% vs. 84.1% (OR 0.99; 95% CI 0.59–1.68, *p* = 0.976); powered (483/483 patients to achieve 80% power, 2-sided alpha 0.05; effect size 8.5%)
Körper et al. August 2021 CAPSID trial [22] (Germany)	Open-label, multicenter RCT	N = 105 hospitalized patients; arm 1: CP + SOC (53); arm 2: SOC (52). 7 patients crossed over to arm 1 due to progressive COVID−19 on day 14.	59 years (IQR 53, 65); solid tumor (3.8% vs. 5.8%); unvaccinated for SARS-CoV-2 (100%)	Glucocorticoids used (84.9% vs. 94.2%); O_2_ used (92.4% vs. 94.3%); other: remdesivir, tocilizumab, antibiotics, vasopressors, anticoagulation, platelet aggregation inhibitor	7 days (IQR 2, 9)	1:320 (1:80–1:640) with 37/53 (69.8%) with neutralizing antibodies (CP arm) vs. 1:160 (1:80–1:640) with 38/52 (73.8%) with neutralizing antibodies (SOC arm)	846 mL(IQR 824, 855); mixture of high and non-high titer CP; (median PRNT50 1:160; IQR 1:80, 1:640)	No significant difference in survival and no longer filling criteria for severe COVID−19 on day 21 in intention to treat analysis: 43.4% vs. 32.7% (*p* = 0.32)’ powered (105/96 patients to achieve 80% power, 2-sided alpha 0.05; effect size 30%)
Avendaño-Solá et al. September 2021 [23] (Spain)	Open-label, multicenter RCT	N = 350 hospitalized patients; arm 1: CP + SOC (179); arm 2: SOC (171)	63 years (IQR 50–75); cancer (8.4% vs. 6.4%); immunodeficiency (5.6% vs. 6.4%); unvaccinated for SARS-CoV-2 (100%)	Glucocorticoids used (70.9% vs. 71.3%); O_2_ used (13.6% vs. 18.9%); others: anticoagulants, remdesivir, azithromycin	6 days (IQR 4, 7)	48/179 (26.8%) positive for SARS-CoV-2-IgG antibodies (CP arm) vs. 61/171 (35.7%) positive for SARS-CoV-2-IgG antibodies (SOC arm)	250–300 mL mixture of high and non-high titer CP (median anti-SARS-CoV-2-IgG antibody anti-S (ORTHO) 8.2; IQR 4.5, 12. 36.6% received “high-titer CP”)	No significant difference in proportion of patients at 14 days who were on noninvasive ventilation, mechanical ventilation, extracorporeal membrane oxygenation, or death in intention to treat analysis: 11.7% vs. 16.4% (*p* = 0.21); powered 350/278 patients to achieve 80% power, 1-sided alpha 0.025, effect size 10%.
Bégin et al. September 2021 CONCOR-1 trial [24] (USA, Canada, Brazil)	Open-label, multicenter RCT	N = 921 hospitalized patients; arm 1: CP + SOC (614); arm 2: SOC (307)	67.7 years (IQR 58, 80); no patients specified with cancer or immunosuppressant medication; unvaccinated for SARS-CoV-2 (100%)	Glucocorticoids used (79.4% vs. 82.4%); FiO_2_ at baseline: 40 (IQR 30, 65); other medications: azithromycin, antiviral medications, anticoagulants	8 days (IQR 5, 10)	N/A	500 mL mixture of high and non-high titer CP (PRNT50 at least 1:160 or antibody against RBD of SARS-CoV-2 spike protein titer of 1:100)	No significant difference in intubation or death in intention to treat analysis: 32.4% vs. 28% (RR 1.16, 95% CI 0.94–1.43, *p* = 0.18); underpowered (921/1200 patients to achieve 80% power, 2-sided alpha 0.05, effect size 25%). Trial stopped early at planned interim analysis for futility because conditional power estimate was 1.6% (below stopping criterion of 20%)
Estcourt et al. November 2021 REMAP-CAP [25] (UK, Canada, USA, Australia)	Open-label, multicenter RCT	N = 2011 critically ill (ICU) hospitalized patients; arm 1: 2 units CP + SOC (1095); arm 2: SOC (916)	61 years (IQR 52,70); immunosuppressive disease or therapy (6.3% vs. 6.6%); unvaccinated for SARS-CoV-2 (100%)	Glucocorticoids used (94.1% vs. 93%); O_2_ used 100%; other medications: remdesivir, tocilizumab or sarilumab.	N/A	603/874 (69%) positive for SARS-CoV-2-IgG antibodies (CP arm) vs. 409/558 (73%) positive for SARS-CoV-2-IgG antibodies (SOC arm)	550 mL ± 150 mL high-titer CP (99% had EUROIMMUN titers greater than 6)	No significant difference in organ support-free days: 0 (IQR −1 to 16) vs. 3 (IQR, −1 to 16) (OR 0.97, 95% credible interval 0.83 to 1.15). Trial stopped early as futility was met (posterior probability of futility OR < 1.2 was 99.4%).
Menichetti et al. November 2021. TSUNAMI study group [26] (Italy)	Open-label, multicenter RCT	N = 487 hospitalized patients; arm 1: CP + SOC (241); arm 2: SOC (246)	64 years (IQR 54,74); solid tumors (4.3% vs. 2.9%); unvaccinated for SARS-CoV-2 (100%)	Glucocorticoids used (19.4% vs. 21.6%); other: remdesivir, low molecular weight heparin.	7 days (IQR 5–9)	N/A	200 mL (1–3 transfusions); high titer (at least 1:160 by microneutralization test)	No significant difference in composite endpoint of worsening respiratory failure: 25.5% vs. 28% (OR 0.88, 95% CI 0.59–1.33, *p* = 0.54); powered: 487/474 patients enrolled to achieve 80% power, 2-sided alpha 0.05, effect size 40%.
Bar et al. December 2021 [27] (USA)	Open-label, single health system RCT	N = 80 hospitalized patients; arm 1: CP + SOC (40); arm 2: SOC (39)	63 years (IQR 52,74l); immunodeficiency (12.5% vs. 15.4%); cancer (25% vs. 28.2%); unvaccinated for SARS-CoV-2 (100%)	Glucocorticoids used (77.5% vs. 89.7%); other: remdesivir, hydroxychloroquine	6 days (IQR 4–9)	32/79 (40%) were positive for SARS-CoV-2-IgG antibodies in entire cohort	Up to two units of CP were allowed (ml not specified); mixture of low and high titer (62% plasma received high titer)	Improved clinical severity score 10 (5.5–30) vs. 7 (2.75–12.25), *p* = 0.037. Powered (80/80 patients needed to achieve 80% power to reject null proportion 50% if experimental treatment with 80% or higher probability of having better severity than control participant)
Berg et al. February 2022. PROTECT-Patient [28] (South Africa)	Double-blinded, multicenter, placebo controlled RCT	N = 103 hospitalized patients; arm 1: CP + SOC (52); arm 2: saline + SOC (51)	56 years (IQR 46,63); HIV positive (11.5% vs. 28.8%); unvaccinated for SARS-CoV-2 (100%)	Glucocorticoids used (96.2% vs. 92.2%); O_2_ use: 100%; other: anticoagulation.	8 days (IQR 6–11)	N/A	200–250 mL high titer (neutralizing antibody titers of 1:160 or higher)	No significant difference in hospital discharge or improvement in WHO blueprint ordinal scale for clinical improvement (RR 1.03, 95% CI 0.77–1.38); underpowered (103/600 patients to achieve 80% power, 20-sided alpha 0.05, effect size 33%). Study stopped enrolling due to futility
Ortigoza et al. February 2022 CONTAIN COVID19 [29] (USA)	Double-blinded, multicenter, placebo-controlled RCT	N = 941 hospitalized patients Arm1: CP + SOC (468) Arm 2: SOC (473)	63 years (IQR 52,73); cancer (11.5% vs. 11.0); transplant (2.4 vs. 0.8%); HIV and immunodeficiency (1.3% vs. 1.3%); unvaccinated for SARS-CoV-2 (100%)	Glucocorticoids used (76.1% vs. 77.2%); requiring O_2_: 100%	7 days (IQR 4–9)	228/468 (64.4%) were positive for SARS-CoV-2 IgG antibodies (CP arm) vs. 258/473 (68.8%) were positive for SARS-CoV-2 IgG antibodies (SOC arm)	250 mL mixture of low and high titer (about 170/941, 18% received high-titer CCP >1:160)	No significant difference in cumulative adjusted odds ratio in 11-point WHO ordinal scale for clinical improvement (cOR 0.94, 95% credible interval 0.75–1.18, posterior probability P[cOR < 1] =72%). Trial stopped recruiting based on 0.2% probability that study would meet criteria for success if enrollment continued to 1000 participants.
Alemany et al. March 2022 CONV-ERT [30] (Spain)	Double-blinded, multicenter, placebo-controlled RCT	N = 376 outpatients; arm 1 methylene blue treated CP + SOC (188); arm 2: saline placebo + SOC (188)	56 years (IQR 52–62); immunocompromised (3% vs. 2%); unvaccinated for SARS-CoV-2 (100%)	No reported use of glucocorticoids. No baseline use of O_2_.	4.4 days (SD 1.4)	Of 326 tested patients, 23/188 (13% were positive for SARS-CoV-2 IgG antibodies (CP arm) vs. 20/186 (11%) were positive for SARS-CoV-2 IgG antibodies (SOC arm)	250–300 mL mostly high titer (89% of tested units had a SARS-CoV-2 neutralizing ID50 of >1:250)	No significant difference in 28-day hospitalization rate: 12% vs. 11% (RR 1.05, 95% CI 0.78–1.41) or viral load change from baseline to day 7 (−2.41 log10 vs. −2.31 log10); underpowered (376/474 patients to achieve 80% power, a = 0.05, effect size 50%). Trial terminated early because more than 85% of population aged 50 years or older were fully vaccinated in Spain and because monoclonal antibodies were available for outpatients at high risk for progression to severe COVID-19.
Sullivan et al. May 2022 CSSC-004 [31] (USA)	Double-blinded, multicenter, placebo-controlled RCT	N = 1225 outpatients Arm 1: CP (610) Arm 2: Control Plasma (589)	43 years (IQR 32–55); unvaccinated for SARS-CoV-2 (83.3% vs. 81.7%); partially vaccinated for SARS-CoV-2 (4.6% vs. 5.3%); active Cancer (0.5% vs. 0.5%) HIV (2% vs. 2.2%) Immunosuppressed (0.3% vs. 0%)	No glucocorticoids used. No baseline used of O_2_. Other: none.	5 days (IQR 4–7)	N/A	250 mL mixture of high and non-high titer CP (80% of units had SARS-CoV-2 spike protein antibody titers of at least 1:4860 or high titer)	Reduced COVID-19-related hospitalization within 28 days after transfusion (in modified intent to treat analysis including only those who received CP): 3.4% absolute risk reduction (95% CI 1.0–5.8; *p* = 0.005); underpowered (1225/1344 patients to achieve 80% power, 1-sided alpha 0.05, effect size 25%). Trial stopped early after 90% of initial enrollment was reached due to declining numbers of hospitalizations for COVID19

Abbreviations: ELISA = enzyme-linked immunosorbent assay; IQR = interquartile range; N = number; SOC = standard of care; OR = odds ratio; ORTHO = Ortho Clinical Diagnostics, Rochester, New York, USA; PRNT50 = plaque-reduction neutralization test 50% inhibition. RR = relative risk. In August 2020, the FDA Emergency Use Authorization defined high-titer convalescent plasma based on the live-virus, five-dilution plaque reduction neutralization test as a 50% inhibitory dilution (ID_50_) of 1:250 or more. The FDA EUA also considers Ortho VITROS Anti SARS-CoV-2 IgG S/C ≥ 9.5, EUROIMMUN Anti-SARS-CoV-2 ELISA ratio ≥ 3.5 as high-titer [10].
